# Morbidity and mortality in small for gestational age very preterm infants in a middle-income country

**DOI:** 10.3389/fped.2022.915796

**Published:** 2022-08-09

**Authors:** Marcia Mangiza, Danielle E. Y. Ehret, Erika M. Edwards, Natasha Rhoda, Lloyd Tooke

**Affiliations:** ^1^Groote Schuur Hospital, Department of Paediatrics, University of Cape Town, Cape Town, South Africa; ^2^Vermont Oxford Network, Burlington, VT, United States; ^3^Department of Paediatrics, Larner College of Medicine, University of Vermont, Burlington, VT, United States; ^4^Department of Mathematics and Statistics, University of Vermont, Burlington, VT, United States

**Keywords:** small for gestational age, low- and middle-income countries, very low birth weight (VLBW), bronchopulmonary dysplasia, late onset sepsis, preterm

## Abstract

**Objective:**

To evaluate the impact of small for gestational age (SGA) on outcomes of very preterm infants at Groote Schuur Hospital (GSH), Cape Town, South Africa.

**Study design:**

Data were obtained from the Vermont Oxford Network (VON) GSH database from 2012 to 2018. The study is a secondary analysis of prospectively collected observational data. Fenton growth charts were used to define SGA as birth weight < 10th centile for gestational age.

**Results:**

Mortality [28.9% vs. 18.5%, adjusted risk ratio (aRR) 2.1, 95% confidence interval (CI) 1.6–2.7], bronchopulmonary dysplasia (BPD; 14% vs. 4.5%, aRR 3.7, 95% CI 2.3–6.1), and late-onset sepsis (LOS; 16.7% vs. 9.6%, aRR 2.3, 95% CI 1.6–3.3) were higher in the SGA than in the non-SGA group.

**Conclusion:**

Small for gestational age infants have a higher risk of mortality and morbidity among very preterm infants at GSH. This may be useful for counseling and perinatal management.

## Introduction

Small for gestational age (SGA) is defined as a birth weight that is below the 10th percentile for gestational age (1). SGA includes infants who are constitutionally small and those who have intrauterine growth restrictions due to environmental or genetic factors (2).

Small for gestational age is commonly used as a proxy for intrauterine growth restriction and, in settings with a high prevalence of SGA, it is more likely to be a result of intrauterine growth restriction rather than being constitutionally small (3). Intrauterine growth restriction is more prevalent in low- and middle-income countries (LMICs) (4), due to contributing factors, such as poor maternal nutrition, young maternal age, maternal infections, and short birth spacing (5–8). Approximately one in five infants born in LMICs is SGA (4). Despite this high prevalence of SGA, the outcomes of preterm babies have not been well described in these settings.

In LMICs, mortality risk associated with being premature and SGA is substantially higher than for either alone, with these infants having a 10–40 times higher risk of mortality in the first month of life when compared with term and appropriate for gestational age infants (9). We sought to evaluate the impact of SGA on the outcomes of very low birth weight (VLBW) and very preterm (<32 weeks) infants at the Groote Schuur Hospital (GSH) neonatal unit in Cape Town, South Africa. Our objectives were to compare rates of in-hospital mortality and neonatal morbidities of SGA preterm infants with non-SGA preterm infants and to compare outcomes of symmetrical vs. asymmetrical infants with SGA.

## Materials and methods

The study is a secondary analysis of prospectively collected observational data. GSH is a public hospital with a highly specialized public neonatal unit. There are 75 neonatal beds of which 20 are for intensive care. The neonatal unit admits up to 2,000 babies a year and approximately 500 infants are VLBW. We obtained data from the Vermont Oxford Network (VON) GSH database from 2012 (when we joined the Network) to 2018. VON is a non-profit, voluntary worldwide collaboration dedicated to improving the quality, safety, and value of neonatal intensive care (10). The database includes items, such as respiratory support in the delivery room, in the neonatal intensive care unit, and at discharge; other procedures and interventions, such as surgery; morbidities and mortality; length of stay; infant and maternal characteristics; and status at discharge. The GSH VON VLBW database enrolls neonates with birth weights between 401 and 1,500 g if they are born at GSH or transferred there within the first 28 days of life. Infants that are subsequently transferred from GSH to other hospitals are followed up to document mortality and mortality prior to discharge in the GSH VON VLBW database. Our study included infants born from 27 weeks 0 days to 30 weeks 6 days gestational age in order to capture both SGA and appropriate for gestational age (AGA) for the weight category 501–1,500 g. We excluded neonates with major congenital or chromosomal anomalies. The study was approved by the Human Research Ethics Committee of the Health Sciences Faculty of the University of Cape Town (R117/2020).

### Study variables

Small for gestational age was defined as a birth weight below the 10th centile and non-SGA as birth weight ≥the 10th centile using sex-specific Fenton growth charts (11). Non-SGA comprised of AGA was defined as between the 10th and 90th centile and large for gestational age (LGA) was defined as >90th centile on the sex-specific Fenton growth charts. Gestational age was estimated using early ultrasound (<20 weeks) as the gold standard. Ballard score or postnatal foot length (12) were used when early ultrasound was not available. Symmetric SGA was defined as both birth weight and head circumference below the 10th centile on the Fenton growth chart and asymmetric SGA when birth weight was below the 10th centile and head circumference was above the 10th centile for gestational age on the Fenton growth chart.

Infants were considered to have exposure to antenatal corticosteroids if betamethasone, dexamethasone, or hydrocortisone was administered intramuscularly or intravenously to the mother during pregnancy at any time prior to delivery. Mothers were reported to have received prenatal care if any obstetric care was provided prior to the admission during which the birth occurred. Maternal hypertension was defined as chronic or pregnancy-induced hypertension (above 140 systolic or 90 diastolic), with or without edema or proteinuria. Eclampsia and pre-eclampsia were considered the forms of pregnancy-induced hypertension.

Mortality was defined as death before discharge home. Respiratory distress syndrome (RDS) was defined as respiratory distress from birth with the need for >35% Fio_2_ in the first 48 h of life despite continuous positive airway pressure (CPAP) or invasive mechanical ventilation. Papile’s criterion (13) was used to define intraventricular hemorrhage (IVH) noted on cranial ultrasound scan and severe IVH was defined as grades 3 and 4. Bronchopulmonary dysplasia (BPD) was defined as the requirement of oxygen and/or respiratory support at 36 weeks post-menstrual age or, if discharged at 34 or 35 weeks, oxygen requirement at discharge (14). Severe retinopathy of prematurity (ROP) was defined as grades 3–5 (15) documented by an ophthalmologist’s examination, with the worst stage examination in the eye with the most advanced stage recorded. Early onset sepsis was defined as recovery of a bacterial organism on a specified list from blood or cerebrospinal fluid culture within 3 days of birth. Late-onset sepsis (LOS) was defined as recovery of a bacterial organism on a specified list from blood or cerebrospinal fluid culture, coagulase negative staphylococcal infection, or fungal organism after day 3 from birth; coagulase negative staphylococcus also required signs and symptoms of infection and at least 5 days of antibiotic therapy (16). Necrotizing enterocolitis (NEC) was diagnosed by the clinical team at surgery, post-mortem, or clinically and radiographically using standard criteria from the VON Manual of Operations Definitions (16).

### Analyses

All analyses were conducted in Statistical Analysis System 9.4. Logistic regression with a Poisson distribution and log link was used to produce risk ratios for each outcome adjusted for sex, antenatal steroid exposure, inborn/outborn status, and gestational age in weeks (17). All adjustors, identified *a priori*, were included in each model without stepwise or backward selection.

## Results

### Characteristics of the study population

**FIGURE 1:**
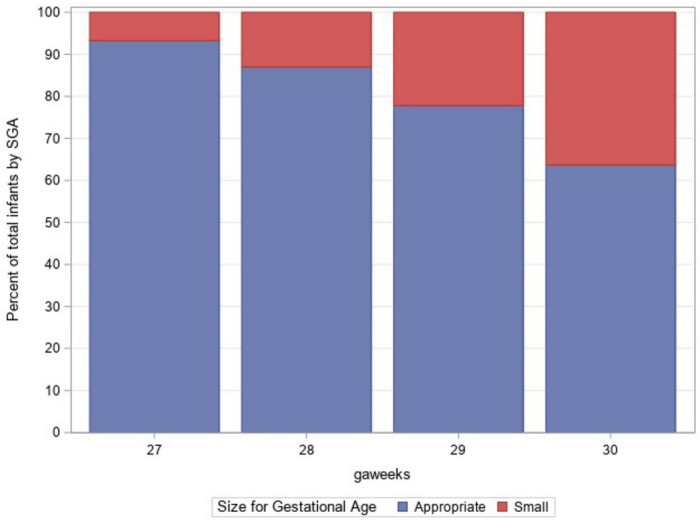
Percent of total infants by small for gestational age (SGA; red) and non SGA (blue).

A total of 1,879 infants with VLBW between the gestational ages of 27 weeks 0 days and 30 weeks 6 days were admitted to GSH NICU between 2012 and 2018. The prevalence of SGA among the study population was 12.7%. The mean gestational age for both infants with SGA and non-SGA was 29 weeks. The mean birth weight in the SGA group was 799 and 1,083 g in the non-SGA group. The majority (99%) of the non-SGA group was AGA with only 20 infants being LGA. The mothers of infants with SGA were more likely to have received antenatal steroids (73.6% vs. 66.4%) and to have hypertension (60.1% vs. 40.4%). Table 1 depicts the demographic features of the study population based on SGA status. Figure 1 shows the proportion of infants with SGA at each gestational age.

**TABLE 1:** Demographic features of the study population of infants who were born 27–30 weeks + 6 days gestational age and 501–1,500 g from 2012 to 2018.

	SGA (*N* = 239)	Non-SGA (*N* = 1640)
Gestational age (weeks), mean (SD)	29 (1)	29 (1)
Birth weight (grams), mean (SD)	799 (119)	1083 (182)
Head circumference at birth (cm), mean (standard deviation)	24 (2)	26 (2)
Maternal hypertension (%)	60.1	40.4
Antenatal steroids (%)	73.6	66.4
Female (%)	52.7	48.4
Prenatal care (%)	87.5	82.4
Inborn (%)	91.2	85.4

SD, standard deviation.

### Neonatal morbidity and mortality

During the study period, 372 of the infants demised before being discharge home with an overall mortality rate of 19.7%. The mortality rate was significantly higher in the SGA group at 28.9% vs. 18.5% [adjusted risk ratio (aRR) 2.1, 95% confidence interval (CI) 1.6–2.7] when compared to the non-SGA group. BPD, NEC, and LOS were significantly higher in the SGA group. Table 2 shows the incidence of neonatal morbidity and mortality in SGA vs. non-SGA VLBW neonates.

**TABLE 2:** Incidence of neonatal morbidity and mortality in very low birth weight infants classified as small for gestational age (SGA) or appropriate for gestational age (AGA). Risk ratios for the association between SGA and non-SGA (reference group) adjusted for sex, antenatal steroids, and gestational age at birth.

	SGA (*N* = 239)	Non-SGA (*N* = 1640)	aRR (95% CI)
	*n*/*N* (%)	*n*/*N* (%)	
MORTALITY	69/239 (28.9)	303/1640 (18.5)	2.1 (1.6, 2.7)
RDS	103/238 (43.3)	764/1632 (46.8)	1.0 (0.8, 1.3)
BPD	24/171 (14.0)	61/1343 (4.5)	3.7 (2.3, 6.1)
IVH (Grades III, IV)	10/211 (4.7)	116/1512 (7.7)	0.8 (0.4, 1.6)
NEC	24/238 (10.1)	107/1632 (6.6)	1.7 (1.1, 2.7)
ROP (Stages III–V)	4/78 (5.1)	15/579 (2.6)	3.2 (1.0, 10.2)
Early onset sepsis	2/238 (0.8)	30/1632 (1.8)	0.5 (0.1, 2.1)
Late onset sepsis	36/216 (16.7)	146/1522 (9.6)	2.3 (1.6, 3.3)

RDS, respiratory distress syndrome; BPD, bronchopulmonary dysplasia; IVH, intraventricular hemorrhage; NEC, necrotizing enterocolitis; ROP, retinopathy of prematurity.

Table 3 shows the incidence of neonatal morbidities among survivors in the study population. Among survivors, only BPD and LOS remained statistically significant, with both morbidities occurring more frequently in the SGA group. The total length of stay among survivors was a median of 42 days [interquartile range (IQR) 28, 56] for infants with SGA and a median of 41 days (IQR 25, 56) for infants with non-SGA.

**TABLE 3:** Incidence of neonatal morbidities among survivors in very low birth weight infants classified as small for gestational age (SGA) or non-SGA. Risk ratios for the association between SGA and appropriate for gestational age (AGA; reference group) adjusted for sex, antenatal steroids, and gestational age at birth.

	SGA (*N* = 170)	Non-SGA (*N* = 1337)	aRR (95% CI)
	*n*/*N* (%)	*n*/*N* (%)	
RDS	53/170 (31.2)	538/1336 (40.3)	0.9 (0.7, 1.2)
BPD	24/170 (14.1)	56/1328 (4.2)	4.0 (2.5, 6.7)
IVH (Grades III, IV)	1/168 (0.6)	45/1280 (3.5)	0.2 (0.0, 1.6)
NEC	10/170 (5.9)	54/1336 (4.0)	1.7 (0.9, 3.5)
ROP (Stages III–V)	3/77 (3.9)	15/568 (2.6)	2.5 (0.7, 9.2)
Early onset sepsis	2/170 (1.2)	16/1336 (1.2)	1.1 (0.2, 5.0)
Late onset sepsis	26/170 (15.3)	86/1331 (6.5)	3.3 (2.1, 5.2)

Among the infants with SGA, 217 were categorized into symmetrical (97; 45%) and asymmetrical (120; 55%) SGA (22 infants were missing birth head circumference). There were no statistically significant differences in mortality or morbidities when these two groups were compared.

## Discussion

In our study population, 12.7% of the infants were SGA as defined as weight less than the 10th centile at birth by the Fenton growth charts (11). This is higher than the prevalence in two studies done in high-income countries, which found an SGA prevalence of 9% among infants 501–1,500 g (18) and 10% among infants 22–29 weeks (19). Our finding is in support of studies that have documented SGA to be more prevalent in resource-limited countries (4).

We found the mortality rate to be significantly higher among infants with SGA as compared to infants with non-SGA. The increased risk of mortality is similar to the mortality that Li-Yi Tsai et al. found in Taiwan, which was a middle-income country at that time (20). Overall mortality rate in the Taiwanese study was less than our study (14.7% vs. 19.7%), although the Taiwanese study did include infants till 32 weeks gestation. Several studies in high-income countries have evaluated the association of SGA status with the outcomes of infants with VLBW. These analyses have differing conclusions depending on the basis of the comparison groups, by gestational age at birth or by birth weight. In a study including VON member hospitals in the United States of America (United States), Boghossian et al. found an increased risk of mortality among infants with SGA born at 22–29 weeks’ gestational age and they noted that mortality was, however, not homogenous across gestational ages (19). In contrast to our findings, Horbar et al. found that SGA status was associated with an increased likelihood of survival among infants with VLBW in the first 28 days at participating VON member hospitals in the United States (21). The analyses in this study, however, were based on birth weight rather than gestational age. The authors concluded that SGA had a lower risk of mortality than AGA at any given birth weight, recognizing that these infants were more mature, with older gestational ages at birth. This distinction in analyses is important as gestational dating of pregnancies in LMIC is improving (22), allowing for better characterization of preterm risks. Our study, therefore, compared infants based on gestational age using a hierarchy of early ultrasound, followed by postnatal examination with Ballard score or foot length. In infants with extremely low birth weight (ELBW) in United Kingdom, Charles et al. found no difference in mortality between SGA and AGA even after correcting for gestational age (23). Their study population was more premature than ours with generally higher mortality. Although the ELBW population is a high-risk group globally, they do not represent the focus of neonatal improvement efforts in LMICs, hence the VLBW inclusion in our study.

Levels of BPD are very low at GSH (24) but we found an increased risk of BPD among infants with SGA, 14% vs. 4.5% in the non-SGA group. This finding is in keeping with other studies, which have found the risks of BPD to be 2- to 6-fold higher among infants with SGA (23, 25, 26). Proposed mechanisms for the increased risk of BPD include exposure to pro-inflammatory cytokines both prenatally and immediately postnatally in addition to malnutrition (27). Despite having an increased risk of BPD, there was no statistically significant difference in RDS between the infants with SGA and non-SGA. It is postulated that in infants with SGA, the incidence of RDS is much lower because of increased corticosteroid production as a result of exposure to prenatal stress (28). Despite a higher percentage of antenatal steroids in the SGA group as compared to the AGA group, the incidence of RDS was not lower in the infants with SGA. Our clinical definition of RDS could have captured additional respiratory pathology unrelated to surfactant deficiency, such as congenital pneumonia and respiratory distress due to sepsis, and therefore masked a true difference in RDS. However, in a study including US member hospitals of VON, Boghossian et al. found no difference in the incidence of RDS between infants with SGA and non-SGA who had not received antenatal steroids and for those who received antenatal steroids, the incidence of RDS was higher among the SGA group (19).

Necrotizing enterocolitis is the most serious gastrointestinal complication affecting infants with VLBW. The risk of NEC was higher in the SGA group when looking at the entire study population but when we analyzed the risk among survivors the difference between the groups did not reach statistical significance. This finding suggests a high mortality for SGA babies who develop NEC. Bhoghossian et al. also found an increased risk of NEC among infants with SGA (19). Redistribution of blood flow with a resultant reduction of blood flow to the splanchnic arteries is thought to be some of the mechanisms that increase the risk of NEC in growth-restricted infants (29).

Infants with SGA in our study were more prone to developing late-onset neonatal sepsis. Troger et al. had similar findings and this was elucidated by the increased use of central venous catheters and a longer time to reach enteral feeds in the SGA population (30). Previous studies have noted thymic atrophy, as well as lymphopenia and deficiencies in humoral responses, in infants with SGA as additional potential mechanisms related to LOS (31).

As more premature infants survive due to improved neonatal care, the incidence of ROP has been increasing and South Africa has become a part of the third epidemic of ROP (32). Bhoghossian et al. (19) in the VON United States study and Tsai et al. (20) in Taiwan found an increased risk of ROP among infants with SGA. We did not find a statistically significant risk of ROP in our SGA babies. This may be explained by the small numbers of infants who were screened as the screening program for ROP only started in 2015 at GSH.

A limitation of this study is that only 30–40% of infants in our institution have an early ultrasound scan (<20 weeks’ gestation). When an early ultrasound was not available, Ballard score or postnatal foot length was used to estimate gestational age, which may be less accurate by up to 2 weeks (33). Other limitations include that our study was a single-site hospital study hence it may not be generalized to all resource-limited settings. We also only looked at in-hospital mortality and yet the associations of SGA may be long term. This study is one of the very few studies to report on outcomes of SGA VLBW outcomes in a middle-income country. The study was done in a tertiary setting with high volumes of VLBW. We used the VON database with standard data definitions adapted to harmonize with institutional guidelines in management.

## Conclusion

Groote Schuur Hospital, a public academic hospital in a middle-income country, had a 12.7% rate of infants with SGA VLBW, higher than often reported in high-income countries. Infants with SGA VLBW at GSH had approximately two times the risk of mortality as compared to their AGA counterparts and increased morbidities. These findings are useful for perinatal decision-making and counseling of parents.

## Data availability statement

The raw data supporting the conclusions of this article will be made available by the authors, without undue reservation.

## Ethics statement

This study was approved by the Human Research Ethics Committee of the Health Sciences Faculty of the University of Cape Town (R117/2020). Written informed consent from the participants’ legal guardian/next of kin was not required to participate in this study in accordance with the national legislation and the institutional requirements.

## Author contributions

MM was responsible for designing the study proposal, writing the protocol and report, interpreting results, and looking up references. DE was responsible for designing the study proposal, editing the protocol and report, interpreting results, and updating reference list. EE was responsible for designing the study proposal, analyzing the data, designing tables and figure, and editing the report and references. NR was responsible for designing the study proposal and editing the report. LT was responsible for designing the study proposal, editing the protocol and report, interpreting results, designing Figure 1, and updating references. All authors contributed to the article and approved the submitted version.
